# Synthesis and Evaluation of Novel Pyrazole Ethandiamide Compounds as Inhibitors of Human THP-1 Monocytic Cell Neurotoxicity

**DOI:** 10.3390/cells8070655

**Published:** 2019-06-29

**Authors:** Jordan A. McKenzie, Reham F. Barghash, Azhaar T. Alsaggaf, Omkar Kulkarni, Kalun Boudreau, Frederic Menard, Edward G. Neeland, Andis Klegeris

**Affiliations:** 1 Department of Biology, University of British Columbia Okanagan Campus, Kelowna, BC V1V 1V7, Canada; 2 Department of Chemistry, University of British Columbia Okanagan Campus, Kelowna, BC V1V 1V7, Canada; 3 Chemical Research Industries Division, National Research Centre, Dokki, Giza D-12622, Egypt; 4 Department of Chemistry, Taibah University, Medina 42353, Saudi Arabia

**Keywords:** anti-inflammatory drugs, chemical synthesis, cytotoxicity, microglia models, neuroinflammation, neuroprotection, SH-SY5Y neuroblastoma, THP-1 monocytic cells

## Abstract

Neuroinflammation and microglia-mediated neurotoxicity contribute to the pathogenesis of a broad range of neurodegenerative diseases; therefore, identifying novel compounds that can suppress adverse activation of glia is an important goal. We have previously identified a class of trisubstituted pyrazoles that possess neuroprotective and anti-inflammatory properties. Here, we describe a second generation of pyrazole analogs that were designed to improve their neuroprotective activity toward neurons under inflammatory conditions. Pyrazolyl oxalamide derivatives were designed to explore the effects of steric and electronic factors. Three in vitro assays were performed to evaluate the compounds’ anti-neurotoxic, neuroprotective, and cytotoxic activity using human THP-1, PC-3, and SH-SY5Y cells. Five compounds significantly reduced the neurotoxic secretions from immune-stimulated microglia-like human THP-1 monocytic cells. One of these compounds was also found to protect SH-SY5Y neuronal cells when they were exposed to cytotoxic THP-1 cell supernatants. While one of the analogs was discarded due to its interference with the cell viability assay, most compounds were innocuous to the cultured cells at the concentrations used (1–100 μM). The new compounds reported herein provide a design template for the future development of lead candidates as novel inhibitors of neuroinflammation and neuroprotective drugs.

## 1. Introduction

Alzheimer’s disease (AD) is characterized by inflammatory processes driven by non-neuronal cells, such as microglia [1]. As resident immune cells in the brain, microglia belong to the mononuclear phagocyte system and have been found to become activated in AD [2,3]. They can phagocytose and degrade cell debris and pathological protein aggregates, which is generally believed to help maintain homeostatic conditions in the brain. However, adversely activated microglia can become neurocytopathic through excessive release of inflammatory cytokines and chemokines, reactive oxygen and nitrogen species, as well as other cytotoxins [4]. Considerable experimental and epidemiological evidence indicates that inhibiting adverse microglial activation in AD could be neuroprotective [5]. Both steroidal and non-steroidal anti-inflammatory drugs (NSAIDs) have been tested as inhibitors of microglial adverse activation. Unfortunately, these classes of anti-inflammatory medications have not been effective in most clinical trials performed [6]. These setbacks highlight the need to discover novel anti-inflammatory drugs, with modes of action that differ from those of NSAIDs and steroidal anti-inflammatory medicines, as therapeutic options to treat AD [7,8,9].

Drugs containing the pyrazole moiety have been shown to display a wide range of biological activities, including immunosuppressive, anti-inflammatory, and anti-cancer activity [10,11,12,13]. In fact, a reduced secretion of inflammatory cytokines tumor necrosis factor-α (TNF-α) and interleukin (IL)-6 was observed when stimulated monocytic cells were treated with 3,5-diarylpyrazole derivatives [10]. We observed similar effects with the pyrazole-containing drugs omeprazole and lansoprazole, i.e., the neurotoxicity of primary human microglia was reduced [14]. We also found that the neuroprotective effects of related pyrazole derivatives likely occur through inhibition of microglia activation, both in vitro and in vivo [14].

In previous work, we identified a series of pyrazole oxalamides as potentially neuroprotective and anti-inflammatory compounds [14,15,16]. We now report a second generation of pyrazole oxalamides with improved activity: specifically, seven unsymmetrical oxalamides were synthesized, characterized, and tested. The following three in vitro assays were used, which had been utilized previously to demonstrate the biological activity of other pyrazole derivatives [15,16,17]: (1) transfer of cytotoxic supernatants from immune-stimulated human monocytic THP-1 cells to human SH-SY5Y neuroblastoma cells. Human cell lines were chosen since our previous studies showed that this assay modeled the cytotoxic activity of primary human microglia well and that THP-1 cells could be used as microglia models to study the anti-neurotoxic activity of pharmacological agents [18]; (2) assessment of the ability of the newly synthesized compounds to protect SH-SY5Y neuroblastoma cells cells from neurotoxicity induced by stimulated THP-1 cell supernatants [15,18]; (3) evaluation of the direct toxicity of the compounds by using THP-1 monocytic cells as well as human prostate cancer PC-3 cells [15,16,17]. The results obtained indicate potential structure–activity relationship elements that could be exploited to develop lead drug candidates.

## 2. Materials and Methods

### 2.1. Chemical Synthesis

All commercially available reagents were procured from Sigma–Aldrich (St. Louis, MO, USA), Fisher Scientific (Ottawa, ON, Canada), or Alfa Aesar (Heysham, UK). Reaction solvents were dried by storage over 3 Å molecular sieves. NMR spectra were acquired on a 400 MHz Varian NMR AS400 unit equipped with an ATB-400 probe at 25 °C. Infrared (IR) spectra of compounds were obtained using a Nicolet 6700 FT-IR spectrometer or a PerkinElmer FT-IR Spectrum Two IR spectrometer. High-resolution mass spectrometry analyses were recorded with a HCTultra PTM Discovery System or with a Waters Micromass LCT Premier TOF Mass Spectrometer. Melting points of solid samples were measured with an IA9200 melting point apparatus (Electrothermal, Staffordshire, UK). Column chromatography was carried out on silica gel (230–400 mesh, Silicycle, QC, Canada). Thin-layer chromatography (TLC) analysis was done using precoated glass-backed TLC plates (silica gel thickness 250 μm and 60 Å pore size) from VWR International (Mississauga, ON, Canada).

### 2.2. Synthesis of 2-Oxo-2-substituted-N-(4-cyano-1-phenyl-1H-pyrazol-5-yl)acetamides

**Figure 1:**
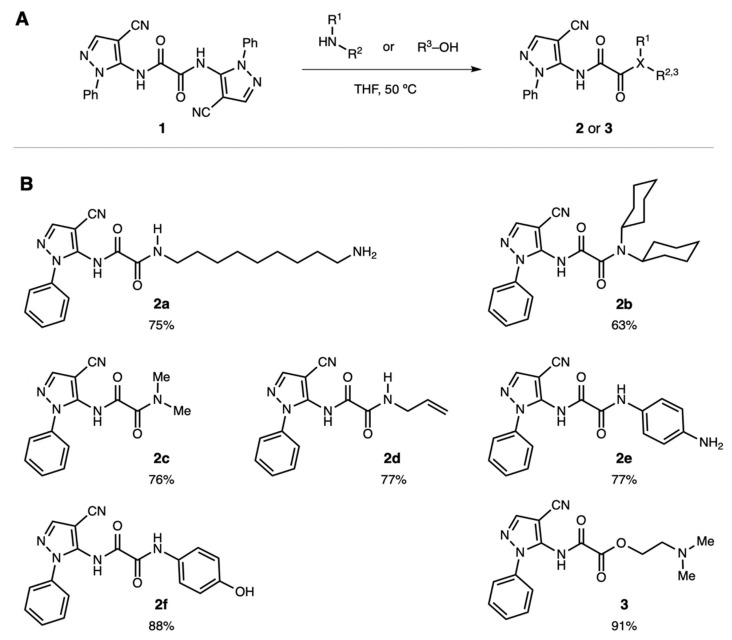
Synthesis of pyrazolyl oxalamides **2** and **3**. (**A**) Reaction conditions used for the monoacylation of oxaladiamide **1**. (**B**) Structures of unsymmetrical oxalamides prepared and evaluated for cytotoxicity and neuroprotective activity. Oxalamides **2a** to **2f** were obtained in 63–88% yield; amidoester **3** was obtained in 91% yield. THF: tetrahydrofuran.

In a flask equipped with a reflux condenser, dipyrazole ethandiamide **1** (0.42 g, 1.0 mmol) was dissolved in 10 mL of tetrahydrofuran (THF) under Ar atmosphere. The primary or secondary amine (4.0 mmol, 4.0 equiv.) was added neat to this solution. The flask was heated to 50 °C for 2 h with constant stirring. The solvent was removed by evaporation under reduced pressure. The crude solids were triturated with 30 mL diethyl ether, filtered in a Buchner funnel (VWR Grade 415 filter paper), washed with 50 mL diethyl ether, and then air-dried. The crystals were washed with diethyl ether and dried under high vacuum. This procedure was followed for the seven oxalamide compounds **2a**–**f** and **3** (see Figure 1 for chemical structures). Complete characterization data for all compounds can be found in Supplementary Materials. For cell culture experiments, pyrazole derivatives were dissolved in pure dimethyl sulfoxide (DMSO) at 20, 10, 2, and 0.2 mM concentrations. The aliquots were added to the cell culture medium directly, for a final DMSO concentration of 0.5%.

### 2.3. Cell Culture

Human PC-3 prostate adenocarcinoma and human THP-1 acute monocytic leukemia cells were obtained from the American Type Culture Collection (ATCC, Manassas, VA, USA). The human neuroblastoma SH-SY5Y cell line was donated by Dr. R. Ross, Fordham University, NY, USA. The cells were grown in Dulbecco’s modified Eagle’s medium–nutrient mixture F12 Ham (DMEM–F12) supplemented with 10% calf bovine serum (CBS), penicillin (100 U mL^−1^), streptomycin (100 μg mL^−1^), and amphotericin B (250 ng mL^−1^). The cell lines were used without differentiation, and the experiments were conducted in DMEM containing 5% CBS.

### 2.4. Measurement of Cell Viability by the 3-(4,5-dimethylthiazol-2-yl)-2,5-diphenyltetrazolium Bromide (MTT) Assay

The MTT assay was performed as described by Mosmann [19] and by Hansen et al. [20]. Cell viability was determined by adding MTT to the cell cultures to reach a final concentration of 500 μg mL^−1^. Following one h incubation at 37 °C, the dark purple crystals formed by viable cells were dissolved by adding to the wells an equal volume of the extraction buffer containing 20% sodium dodecyl sulfate and 50% *N,N*-dimethylformamide, pH 4.7. After overnight incubation at 37 °C, 100 μL aliquots of MTT solutions were transferred to a 96-well plate, and a FLUOstar Omega platereader (BMG Labtech, Offenburg, Germany) was used to measure the optical densities at 570 nm. Cell viability in various samples was calculated as percent of the optical density value obtained with cells incubated in fresh cell culture medium only.

### 2.5. Cytotoxicity of Pyrazole Derivatives

PC-3 prostate cancer cells were transferred to 24-well plates in 500 µL aliquots of 1.5 × 10^5^ cells mL^−1^ per well. Varying concentrations of the pyrazole derivatives or the 0.5% DMSO vehicle were added to the cells. After 48 h incubation, cell viability was measured by using the MTT assay.

### 2.6. Anti-Neurotoxic Activity of Pyrazole Derivatives

The anti-neurotoxic activity of the compounds was evaluated using a method described previously [16]. Human monocytic THP-1 cells were transferred to 24-well plates in 800 µL aliquots of 5 × 10^5^ cells mL^−1^ per well. The cells were pre-incubated with varying concentrations of pyrazole derivatives or their DMSO vehicle solution for 15 min before stimulation with a combination of lipopolysaccharide (LPS, 0.5 µg mL^−1^) and human interferon-γ (IFN-γ, 150 U mL^−1^). Previous studies showed that this combination of stimuli induced maximal pro-inflammatory and cytotoxic response in THP-1 cells and that 15–30 min pre-incubation before stimulation was sufficient to demonstrate the anti-neurotoxic effects of diverse pharmacological agents, including different pyrazole derivatives [15,16,18,21,22,23]. After 24 h incubation, 400 µL of supernatants from THP-1 cells was transferred to SH-SY5Y cells (seeded 24 h earlier in 24-well plates; 400 µL of 2 × 10^5^ cells mL^−1^). The viability of THP-1 cells was measured by the MTT assay immediately after the transfer of the supernatants. The effects of THP-1 cell supernatants on SH-SY5Y neuronal cell viability were measured by the MTT assay after 72 h incubation. The supernatants from unstimulated THP-1 cells did not have a significant effect on SH-SY5Y cell viability, which was comparable to the viability of SH-SY5Y cells incubated in cell culture medium only (data not shown). This indicated that there were sufficient nutrients to support the survival of SH-SY5Y cells during the 96 h incubation period. The concentrations of stimuli and incubation times of THP-1 cells with the stimuli were selected in preliminary experiments so that significant partial killing of SH-SY5Y cells was achieved (20–30% viable cells left), which could be a result of the combined action of cytotoxins secreted by THP-1 cells and depletion of nutrients from the cell culture medium by the stimulated THP-1 cells.

### 2.7. Neuroprotective Activity of Pyrazole Derivatives

SH-SY5Y cells were seeded into 24-well plates (400 µL at 2 × 10^5^ cells mL^−1^). After 24 h incubation, the cell culture medium was replaced with 400 µL of supernatants from THP-1 cells that had been stimulated for 24 h with LPS plus IFN-γ, as described in 2.6. Varying concentrations of the compounds or the DMSO vehicle solution were added directly to SH-SY5Y cells at the time of the supernatant transfer. The viability of SH-SY5Y cells was measured by the MTT assay 72 h later.

### 2.8. Statistical Analysis

Data are presented as means ± standard deviation (SD). Due to considerable variability in the absolute values obtained from independent experiments performed on different days, randomized block design analysis of variance (ANOVA) was used to evaluate the concentration-dependent effects of the compounds, followed by the Dunnett’s post-hoc test; *p* values less than 0.05 were considered statistically significant.

## 3. Results

### 3.1. Synthesis of Pyrazolyloxaladiamide Analogs

A selection of unsymmetrical pyrazolyl oxalamide analogs were synthesized in a single, practical step (Figure 1). Compounds **2** and **3** were synthesized through the monoacylation of the symmetrical bispyrazolyl oxalamide **1** [15]. Under the general conditions shown in Figure 1, a selection of nucleophilic amine or alcohol compounds displaced an aminopyrazole moiety of **1** to provide the unsymmetrical oxalamides **2a**–**f** and amidoester **3** in good to excellent yields (see Supplementary Materials for characterization data). In this acylation reaction, the amines were chosen to explore the steric and electronic effects of substituents on the compounds’ biological activity. Moreover, since these compounds are intended to target cells within the central nervous system, only compounds with logP values appropriate for blood–brain barrier permeation were designed (i.e., logP = 1–4) [24,25]. Accordingly, amides were prepared that bear either aliphatic chains (**2a** and **2d**) or aromatic substituents (**2e** and **2f**). Alternatively, amides with sterically differentiated substituents, including bulky dicyclohexylamide **2b** and smaller dimethylamide **2c,** were prepared. In order to examine potential hydrogen bonding effects, the transacylation reaction was also performed with an alcohol to obtain ester **3**. All logP values for **2** and **3** were found to vary between 1.22 and 3.88, as calculated with ALOGPS 2.1 (www.vcclab.org, see Supplementary Materials).

The new pyrazole derivatives **2a**–**f** and **3** were tested in vitro to determine their cytotoxicity as well as their anti-neurotoxic and neuroprotective properties. Three different human cell lines were selected (THP-1, SH-SY5Y, and PC-3) based on our prior work with a series of pyrazole compounds that displayed promising biological activity [15,16]. The compatibility of compounds **2** and **3** with the assay used in this study was investigated first. Thus, controls were performed with all compounds and the MTT reagent used in the cell viability assay; the absorbance at 570 nm was measured after mixing the compounds with the MTT solution in the absence of cells. At the concentrations used in this study (1–100 µM), only compound **2f** was found to react with the MTT reagent (data not shown); it was therefore excluded from further in vitro studies.

### 3.2. Cytotoxic Effects

**Figure 2:**
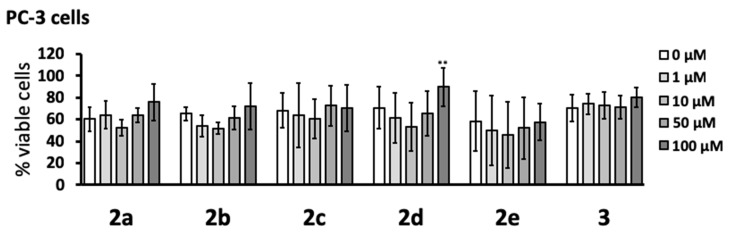
Pyrazole derivatives were not cytotoxic to human PC-3 prostate cancer cells. Compounds **2a**–**e** and **3** were added to PC-3 cells, and their viability was measured 48 h later by the MTT assay. Data (means ± SD) from four independent experiments are presented as percent viable cells. The concentration-dependent effects of the compounds were calculated by randomized block design ANOVA, followed by the Dunnett’s post-hoc test; ** *p* < 0.01 significantly different from PC-3 cells exposed to the dimethyl sulfoxide (DMSO) vehicle only (0 μM).

The cytotoxicity of the pyrazole compounds was determined with human PC-3 prostate cancer cells after 48 h incubation (Figure 2). The MTT assay showed that none of the compounds were cytotoxic at the concentrations studied (1–100 μM). While it was noted that compound **2d** caused a small yet significant increase in cell viability at the highest concentration tested (100 μM), this intriguing effect was not investigated further.

### 3.3. Anti-Neurotoxic Effects

**Figure 3:**
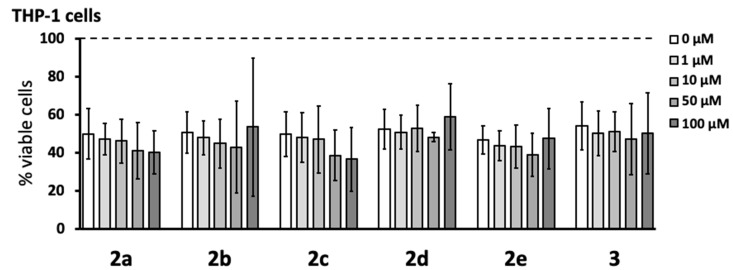
None of the novel pyrazole derivatives reduced the viability of stimulated human monocytic THP-1 cells. Various concentrations (1–100 μM) of pyrazole derivatives were added to THP-1 cells for 15 min before stimulation with lipopolysaccharide (LPS, 0.5 µg mL^−1^) plus interferon-γ (IFN-γ, 150 U mL^−1^). After 24 h incubation, THP-1 cell viability was measured by using the MTT assay. Data (means ± SD) from five independent experiments are presented as percent of viable cells, where 100% viability was measured in THP-1 cells exposed to cell growth medium only. The dotted line represents the viability of unstimulated THP-1 cells. The concentration-dependent effects of the compounds were calculated by the randomized block design ANOVA; no significant effects were observed.

Select pyrazole derivatives have been shown to reduce the secretion of neurotoxins by immune-stimulated human microglia-like THP-1 monocytic cells in an assay where the supernatants from these cells are transferred to human SH-SY5Y neuroblastoma cells, and the viability of neuronal cells is measured after a 72 h incubation period [15,16]. The same in vitro assay was used in this study to estimate the anti-neurotoxic potential of the newly synthesized pyrazole derivatives. We first studied the effect of the six newly synthesized compounds on the viability of THP-1 cells stimulated with a combination of LPS and IFN-γ for 24 h. Figure 3 shows that none of the compounds affected the viability or reduced the numbers of stimulated THP-1 cells over the 1–100 μM pyrazole concentration range tested. We noted that the exposure of THP-1 cells to the stimulating agents generally lowered their viability to approximately 40–60% compared to that of unstimulated cells.

**Figure 4:**
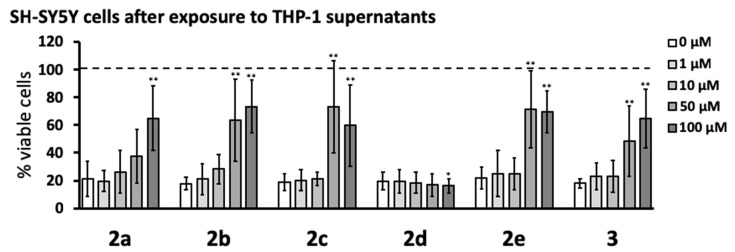
Five pyrazole derivatives reduced the toxicity of THP-1 cells toward SH-SY5Y neuronal cells. THP-1 cells were treated with pyrazole derivatives and stimulated as described in Figure 3 legend. After 24 h incubation, the cell-free supernatants of THP-1 cells were transferred to the wells containing SH-SY5Y neuronal cells. The viability of SH-SY5Y cells was measured after 72 h by the MTT assay. Data (means ± SD) from five independent experiments are presented as percent of viable cells, where 100% viability was measured in SH-SY5Y cells exposed to cell growth medium only. The dotted line represents the viability of SH-SY5Y cells exposed to supernatants from unstimulated THP-1 cells. The concentration-dependent effects of compounds were calculated by the randomized block design ANOVA, followed by the Dunnett’s post-hoc test; * *p* < 0.05, ** *p* < 0.01, significantly different from SH-SY5Y cells exposed to supernatants from THP-1 cells stimulated in the absence of pyrazole compounds (0 μM).

Next, the anti-neurotoxic potential of the novel pyrazole derivatives was studied by measuring the viability of SH-SY5Y cells exposed to supernatants from THP-1 cells stimulated in the absence or presence of 1–100 μM of the compounds. Exposure to supernatants from stimulated THP-1 cells lowered the viability of SH-SY5Y cells to 20–30% compared to that of cells exposed to supernatants from unstimulated THP-1 cells. Figure 4 shows that compounds **2a**, **2b**, **2c**, **2e**, and **3** reduced THP-1 neurotoxic secretions when added 15 min before THP-1 cell stimulation, which increased the viability of SH-SY5Y cells to approximately 60–70%. These effects were statistically significant for **2b**, **2c**, **2e**, and **3** at 50 and 100 μM, while **2a** showed significant protection only at 100 μM. Compound **2d** had no protective effect and at 100 μM, caused a small but significant decrease in SH-SY5Y cell viability. The protective effects of pyrazole derivatives were also studied by measuring the release of lactate dehydrogenase (LDH) into the cell culture medium from dying SH-SY5Y cells after their exposure to supernatants from stimulated THP-1 cells that had been treated with these compounds (see Supplemental Figure 1, Supplementary Materials). Cells undergoing apoptosis during the initial prelytic stages release only low levels of LDH, which leads to underestimation of cell death and inferior sensitivity of the LDH assay compared to the MTT assay (compare Supplemental Figure 1 and Figure 4).

### 3.4. Neuroprotective Effects

**Figure 5:**
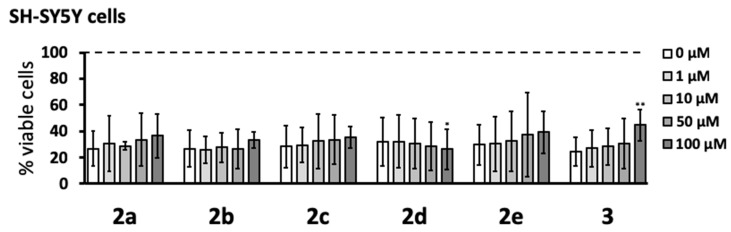
Five pyrazole derivatives did not protect SH-SY5Y cells from toxicity induced by supernatants from stimulated THP-1 cells. Varying concentrations (1–100 μM) of pyrazole derivatives were added directly to SH-SY5Y cells at the time of transfer of the supernatants from THP-1 cells, which were stimulated as described in Figure 3 legend. SH-SY5Y cell viability was measured 72 h later by the MTT assay. Data (means ± SD) from four independent experiments are presented as percent viable cells, where 100% viability was measured in SH-SY5Y cells exposed to cell growth medium only. The dotted line represents the viability of SH-SY5Y cells exposed to supernatants from unstimulated THP-1 cells. The concentration-dependent effects of the compounds were calculated by the randomized block design ANOVA, followed by the Dunnett’s post-hoc test; * *p* < 0.05, ** *p* < 0.01, significantly different from SH-SY5Y cells exposed to stimulated THP-1 supernatants in the absence of pyrazole compounds (0 μM).

The protective effects of pyrazole derivatives on SH-SY5Y cells described above could be caused by the inhibition of THP-1 cells secretions or by a direct protective action of the compounds transferred to the neuronal cells with THP-1 cell supernatants. Consequently, these potential neuroprotective properties were also studied by direct addition of the pyrazole derivatives to SH-SY5Y cells at the time of transfer of the supernatants from stimulated THP-1 cells. As observed in the previous experiment, Figure 5 demonstrates that exposure of SH-SY5Y cells to the supernatants of stimulated THP-1 cells in the absence of pyrazole derivatives reduced their viability to 20–30%. Only compound **3** at the highest concentration studied (100 μM) significantly increased the viability of SH-SY5Y cells above 40%. Similar to the previous experiment, **2d** at 100 μM induced a small but significant decrease in SH-SY5Y cell viability.

## 4. Discussion

The pyrazole derivatives **2** and **3** reported herein are part of a second generation of compounds that builds upon prior work [15,16]. Specifically, we previously found that two series of novel pyrazole derivatives exhibited anti-inflammatory and neuroprotective activity. However, some of these compounds were also cytotoxic, thereby diminishing their promise as neuroprotective drugs. Accordingly, by using human THP-1 monocytic cells as a model, we aimed to discover new molecules that could be used to inhibit adverse microglial activation and that would not be cytotoxic.

New pyrazole oxalamide derivatives were created to identify categories of substituents that display the desired biological activity. Such lead compounds would provide important insight for further structural optimization. Microglia-like human monocytic THP-1 cells were activated by potent immune stimuli. As a response to this activation, these microglia-like cells secreted cytotoxins into their supernatant medium that became toxic to human neuronal SH-SY5Y cells.

A panel of cellular assays was performed with six of the new monopyrazoles: **2a**, **2b**, **2c**, **2d**, **2e**, and **3** (see Figure 1). None of these six derivatives were toxic to THP-1 monocytic cells at concentrations up to 100 µM (see Figure 3). This tolerance was a marked improvement compared to that of the previous series of structurally related pyrazoles, where five out of seven compounds were cytotoxic over the same concentration range [15]. Similarly, cytotoxic effects at concentrations below 100 µM have been reported for several other structurally different pyrazole derivatives [10,15,26]. We further confirmed the lack of cytotoxicity of **2a**–**e** and **3** in a different cell line, human PC-3 prostate cancer cells (see Figure 2).

Compound **2f** was discarded from the above assays, as its cytotoxicity could not be assessed accurately. Compound **2f** interfered with the MTT assay used to measure cell viability. In this assay, the formation of the ring-opened formazan requires a reductive cleavage of MTT’s tetrazolium ring by nicotinamide adenine dinucleotide (NADH). We posit that the phenol group of **2f** may have perturbed the redox balance of NADH. Alternatively, the nucleophilic phenoxide in **2f** may have reacted with MTT’s tetrazolium nitronium group to negate NADH reduction.

Five of the novel compounds were found to significantly reduce the toxic effect of the supernatants from immune-stimulated THP-1 cells on SH-SY5Y neuroblastoma cells (see Figure 4). The IC_50_ for compounds **2b**, **2c**, **2e**, and **3** in this assay were between 10 and 50 µM, while the IC_50_ for **2a** was between 50 and 100 µM. Compound **2d** caused a small yet significant reduction of SH-SY5Y cell viability at the highest concentration used (100 µM). In the same in vitro assay, four of the first-generation pyrazoles showed an IC_50_ that ranged from 5 to 20 µM [15]. The higher IC_50_ of **2a**, compared to other derivatives reported here, may be due to interference from the hydrophilic and sterically larger nonylamine chain (–(CH_2_)_9_NH_2_). The lack of protective activity observed with compound **2d**, compared to the other analogs reported here, may arise from its degradation under the assay conditions. Indeed, **2d** is unique in possessing an allyl group on an electron-deficient amide (making it susceptible to electrophilic displacement), thus it can participate in undesired side reactions.

The increased viability of SH-SY5Y neuronal cells incubated in supernatants from immune-stimulated THP-1 cells treated with pyrazole compounds, compared to that of cells treated with supernatants from THP-1 cells stimulated in the absence of pyrazole compounds, appeared to be due to their inhibition of THP-1 cell noxious secretions. In this assay, the pyrazole derivatives were added to THP-1 cells before their immune stimulation. The THP-1 cell supernatants were then transferred to SH-SY5Y neuronal cells. Therefore, the increased viability of SH-SY5Y neuronal cells could be caused by either the effect of pyrazoles on THP-1 cells (inhibiting secretion of cytotoxins) or a direct neuroprotective effect on SH-SY5Y cells due to the pyrazoles being transferred along with the supernatants. We excluded the latter by finding no significant protective effects on SH-SY5Y viability when compounds **2a**–**e** were administered to SH-SY5Y neuronal cells only at the time of cytotoxic THP-1 supernatant transfer (see Figure 5).

Interestingly, our results indicated that pyrazolyl oxalamides possessing a strong electrophilic site at the carbonyl distal from the pyrazole moiety could directly protect SH-SY5Y neuronal cells from THP-1 secretions when they were added with the supernatants (Figure 5). In this study, compound **3** was unique in possessing an ester instead of an amide and was the only analog to display a neuroprotective effect, albeit significant only at a high concentration (100 µM). The same direct neuroprotective activity was observed with our first-generation pyrazole derivatives [15]. In our earlier report, the compound that showed this effect was the symmetrical bispyrazolyl oxalamide **1**, i.e., the starting material used herein. The reactive electrophilicity of **1** is evident from the fact that it reacted smoothly with amines to create compounds **2a**–**f, 3**. Similarly, the high reactivity of *alpha*-oxoesters is well established [27]. While a detailed mechanistic investigation is beyond the scope of this report, we surmise that cytotoxic molecules released by stimulated THP-1 cells may be irreversibly deactivated by reacting with electrophiles like compound **3**.

A wide range of structurally and functionally diverse small molecules have been reported to inhibit neurotoxicity of microglia or microglia-like monocytic cells. They include, for example, NSAIDs, 5-lipoxygenase, aldose reductase, and phosphodiesterase inhibitors [22,23]. All these compounds could have beneficial effects in Alzheimer’s and other neurodegenerative diseases characterized by adverse microglial activation leading to neurocytopathy. However, some of these drugs show significant adverse effects (e.g., NSAIDs), which limit their long-term use in elderly patients. Therefore, it is essential to identify new classes of clinically safe, selective microglia inhibitors with a low cytotoxicity profile. This can be achieved by identifying novel lead compounds using high-throughput screening combined with thorough structure–activity relationship studies as, for example, described previously for human toll-like receptor 8 agonists [28,29]. The novel pyrazolyl oxalamides described in this study showed no cytotoxic effects and displayed moderate activity as inhibitors of neurotoxic secretions of microglia-like cells.

While further optimization is required to lower their IC_50_ as inhibitors of microglia neurotoxicity, compounds **2** and **3** provide a structural foundation to the discovery of more potent neuroprotective drugs acting as inhibitors of adverse activation of microglia. Future studies will focus on pyrazole analogs containing smaller substituents possessing electrophilic sites.

## Supplementary Materials

Supplementary materials are available online at https://www.mdpi.com/2073-4409/8/7/655/s1.
