# Supplementary material for: Synthesis and Evaluation of Novel Pyrazole Ethandiamide Compounds as Inhibitors of Human THP-1 Monocytic Cell Neurotoxicity
Source: Cells. 2019 Jun 29;8(7):655. doi: 10.3390/cells8070655 (PMC6679371; doi:10.3390/cells8070655)
Supplement: Supplementary file 1 [file cells-08-00655-s001.pdf]

**Chemical Characterization Data**

2-(9-Aminononyl)-2-oxo-*N*-(4'-cyano-1'-phenyl-*1H*-pyrazol-5'-yl) acetamide (**2a**)

Yield 75 %. Mp 108-110 ˚C. IR (cm$^{-1}$): 3550 (NH2), 3381 (NH), 2216 (CN), 1591 (CO-O). $^{1}$H NMR (δ ppm): 8.15 (br s, 1H, NH, $D_2O$ exchangeable), 8.01 (s, 1H, CH), 7.70-7.31 (m, 5H, ArH), 4.08 (br s, 2H, $NH_2$), 2.53 (m, 2H, $CH_2$), 2.52 (m, 2H, $CH_2$), 1.35-1.30 (m, 4H, $CH_2$), 1.27-1.21 (m, 10H, $CH_2$). $^{13}$C NMR (δ ppm): 163.0, 149.6, 142.3, 138.9, 129.7, 127.7, 123.8, 115.0, 86.3, 41.1, 31.9, 29.3, 29.4, 26.5. HRMS (m/z): [M + H]$^+$ Calculated for $C_{21}H_{28}N_6O_2$, 397.2352; found 397.1887; logP: 3.12.

2-Dicyclohexylamino-2-oxo-*N*-(4'-cyano-1'-phenyl-*1H*-pyrazol-5'-yl) acetamide (**2b**)

Yield 63 %. Mp 188-190 ˚C. IR (cm$^{-1}$): 3270 (NH), 2237 (CN),1682 (CO-N) 1610 (CO-NH). $^{1}$H NMR (δ ppm): 10.70 (br s, 1H, NH, $D_2O$ exchangeable), 8.04 (s, 1H, CH), 7.67-7.32 (m, 5H, ArH), 3.07-3.03 (m, 2H, CH), 1.94-1.92 (m, 4H, $CH_2$), 1.73-1.70 (m, 4H, $CH_2$), 1.60-1.57 (m, 2H, $CH_2$), 1.29-1.05 (m, 10H, $CH_2$). $^{13}$C NMR (δ ppm): 162.3, 147.6, 142.6, 138.9, 129.3, 127.8, 123.7, 114.8, 86.4, 52.6, 29.3, 25.2, 25.0, 24.3. HRMS (m/z): [M + H]$^+$ Calculated for $C_{24}H_{29}N_5O_2$, 420.2400; found 420.2405; logP: 3.88.

2-(Dimethylamino)-2-oxo-*N*-(4'-cyano-1'-phenyl-*1H*-pyrazol-5'-yl) acetamide (**2c**)

Yield 76 %. Mp 250-253 °C. IR (cm$^{-1}$): 3227 (NH), 2235 (CN), 1691 (C=O). $^{1}$H NMR (δ ppm): 11.76 (s, 1H, NH, $D_2O$ exchangeable), 8.35 (s, 1H, CH), 7.61–7.30 (m, 5H, ArH), 3.31 (s, 6H, $CH_3$) ppm. $^{13}$C NMR (δ ppm): 158.2, 143.1, 139.9, 137.7, 130.0, 129.5, 124.3, 124.2, 122.2, 112.8, 90.8. HRMS (m/z): [M + H]$^+$ Calculated for $C_{14}H_{13}N_5O_2$, 283.1069; compound did not ionize under standard conditions; logP: 1.22.

2-Allyl-2-oxo-*N*-(4'-cyano-1'-phenyl-*1H*-pyrazol-5'-yl) acetamide (**2d**)

Yield 77 %. Mp 108–110 °C. IR (cm$^{-1}$): 3229 (NH), 2222 (CN), 1639 (C=O). $^{1}$H NMR (δ ppm): 8.88 (br s, 1H, NH, $D_2O$ exchangeable), 7.93 (s, 1H, CH), 7.93–7.40 (m, 5H, ArH), 6.67 (s, 1H, NH, $D_2O$ exchangeable), 5.80 (m 1H, CH), 5.05 (m 2H, $CH_2$), 3.74 (m, 2H, $CH_2$). $^{13}$C NMR (δ ppm): 159.7, 157.9, 142.5, 138.9, 137.2, 129.4, 128.9, 128.6, 128.3, 126.2, 123.8, 112.4, 90.4, 40.6, 34.3. HRMS (m/z): [M + H]$^+$ Calculated for $C_{15}H_{13}N_5O_2$, 296.1147; found 296.1155; logP: 1.62.

2-(4''-Aminophenyl)-2-oxo-*N*-(4'-cyano-1'-phenyl-*1H*-pyrazol-5'-yl) acetamide (**2e**)

Yield 77 %. Mp 258–261 °C. IR (cm$^{-1}$): 3228 (NH), 2237 (CN), 1693 (C=O). $^{1}$H NMR (δ ppm): 8.18 (s, 1H, CH), 7.67–7.34 (m, 5H, ArH), 6.45 (m, 4H, $CH_2$). $^{13}$C NMR (δ ppm): 146.9, 142.7, 138.7, 138.3, 129.5, 128.2, 123.8, 122.1, 116.7, 114.5, 87.4. HRMS (m/z): [M + H]$^+$ Calculated for $C_{18}H_{14}N_6O_2$, 347.1256; found 347.1210; logP: 2.06.

2-(4''-Hydroxyphenylamino)-2-oxo-*N*-(4'-cyano-1'-phenyl-*1H*-pyrazol-5'-yl) acetamide (**2f**)

Yield 88 %. Mp 237-239 ˚C. IR (cm$^{-1}$): 3320-3180 (OH), 3228 (NH), 2237 (CN), 1694 (NH-C=O). $^{1}$H NMR (δ ppm): 8.42 (s, 1H, NH, $D_2O$ exchangeable), 8.29 (s, 1H, CH), 7.59–7.39 (m, 5H, ArH), 6.53–6.38 (m, 4H, $CH_2$). $^{13}$C NMR (δ ppm): 159.8, 149.6, 142.9, 139.3, 138.2, 129.7, 128.9, 124.0, 116.6, 116.1, 113.6, 89.2. HRMS (m/z): [M + H]$^+$ Calculated for $C_{18}H_{13}N_5O_3$, 348.1097; found 348.1357; logP: 2.36.

2-(*N,N*-Dimethyl-2''-aminoethoxy)-2-oxo-*N*-(4'-cyano-1'-phenyl-*1H*-pyrazol-5'-yl) acetamide (**3**)

Yield 91 %. Mp 139-141 ˚C. IR (cm$^{-1}$): 3275 (NH), 2239 (CN), 1705 (CO-O), 1595 (CO-NH). $^{1}$H NMR (δ ppm): 9.95 (br s, 1H, NH, $D_2O$ exchangeable), 8.08 (s, 1H, CH), 7.66-7.34 (m, 5H, ArH), 3.63 (m, 2H, $CH_2$), 3.01 (m, 2H, $CH_2$), 2.67 (s, 6H, $CH_3$). $^{13}$C NMR (δ ppm): 162.0, 147.7, 142.6, 138.9, 129.4, 128.1,

123.8, 114.9, 86.9, 59.0, 55.9, 43.4. HRMS (m/z): $[M + H]^+$ Calculated for $C_{16}H_{17}N_5O_3$, 328.1409; found 328.1410; logP: 1.51.

**Cell death measurement by the lactate dehydrogenase (LDH) assay**

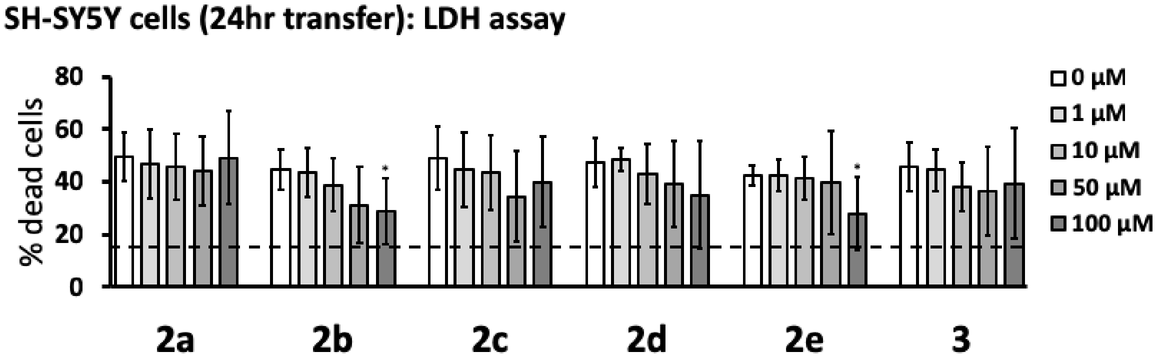


**Supplemental Figure 1:** Two pyrazole derivatives reduced the toxicity of THP-1 cells toward SH-SY5Y neuronal cells according to the lactate dehydrogenase (LDH) assay, which was performed as previously described [15,16]. THP-1 cells were treated with pyrazole derivatives and stimulated as described in Figure 3 legend. After 24 h incubation, the cell-free supernatants of THP-1 cells were transferred to the wells containing SH-SY5Y neuronal cells. Viability of SH-SY5Y cells was measured after 72 h by the LDH assay. Data (means ± SD) from five independent experiments are presented as percent dead cells, where 100% death was achieved by treating SH-SY5Y cells in growth medium only with 1% Triton X-100. Dotted line represents percent dead SH-SY5Y cells exposed to supernatants from unstimulated THP-1 cells. The concentration-dependent effects of compounds were calculated by the randomized block design ANOVA, followed by the Dunnett's *post-hoc* test; * $P < 0.05$, significantly different from SH-SY5Y cells exposed to supernatants from THP-1 cells stimulated in the absence of pyrazole compounds (0 µM).
